# Effect of the consumption of green tea extract during pregnancy and lactation on metabolism of mothers and 28d-old offspring

**DOI:** 10.1038/s41598-018-20174-x

**Published:** 2018-01-30

**Authors:** Ana Claudia Losinskas Hachul, Valter Tadeu Boldarine, Nelson Inácio Pinto Neto, Mayara Franzoi Moreno, Patricia Oliveira Carvalho, Alexandra C. H. F. Sawaya, Eliane Beraldi Ribeiro, Claudia Maria Oller do Nascimento, Lila Missae Oyama

**Affiliations:** 1 Universidade Federal de São Paulo. Escola Paulista de Medicina. Departamento de Fisiologia, São Paulo (SP), Brazil; 2 Laboratory of Multidisciplinary Research, Universidade São Francisco, São Paulo, Brazil; 3 Department of Plant Biology, Universidade Estadual de Campinas, Campinas, São Paulo, 13083970 Brazil

## Abstract

The objective was to investigate the effects of the maternal consumption of the green tea extract during pregnancy and lactation on mothers and offspring metabolism. The female Wistar rats, on the first day of pregnancy until the end of lactation, was divided into groups: MC– received water and ME– received green tea extract (400 mg/kg body weight/day), both ingested control diet. After lactation, at day 28^th^ post-partum, the mothers and pups from each mother were euthanized and composed the groups: FC– pup from mother received water and FE– pup from mother received green tea extract. The ME group increased IL-10/TNF-α ratio and IL-1β content in the mesenteric and IL-1β content in retroperitoneal adipose tissues, and decreased catalase activity. The FE group decreased the retroperitoneal adipose tissue relative weight and SOD activity, but increased adiponectin, LPS, IL-10 and IL-6 content and IL-10/TNF-α ratio in retroperitoneal, IL-10 and TNF-α content in gonadal, and IL-6 content in mesenteric adipose tissues. In summary, the maternal consumption of green tea extract associated with control diet ingestion during pregnancy and lactation altered the inflammatory status of mothers and 28d-old offspring. These data elucidate the effects of green tea during pregnancy and lactation on maternal and offspring metabolism.

## Introduction

Green tea, made from the dried leaves of *Camellia sinensis*, is one of the most popular beverages around the world. The green tea is rich in polyphenols, among them the catechins: epigallocatechin, epicatechin, epicatechin gallate and most abundant, epigallocatechin-3-gallate (EGCG)^1–3^. Epidemiological studies suggest that polyphenolic compounds in the tea reduce the risk of a variety of diseases and present antioxidant properties^4–6^.

The metabolic programming can be defined as a response of the organism to a challenge in developmental period that alters the phenotype with potential persistent effect on health of offspring throughout their lives^7,8^.

However, few studies assess the effects of tea consumption during pregnancy and lactation. One study reported, through *in vitro* rat embryos exposure to the EGCG during early organogenesis, that most of the tea catechins are very safe during early embryonic development^9^. Other studies demonstrated that EGCG was absorbed thoroughly, passed through the placenta, and reached the fetus, where it was distributed to major fetal organs in significant quantities^10,11^.

One study, in rats, investigated potential adverse effects of standardized heat-sterilized green tea catechins (GTC-H) on embryo-fetal development. For this, GTC-H was administered once daily from 6^th^ through 17^th^ day pregnancy and the dosage administered were 0 (control), 200, 600, and 2000 mg/kg/day. The doses used did not affect intrauterine growth and survival fetal malformations or developmental and only the 2000 mg/kg/day dose promoted hair loss in different areas^12^.

The objective of this study was to investigate the effects of the maternal consumption of the green tea extract during pregnancy and lactation on mothers and offspring metabolism.

## Results

### Body weight and delta weight

**Figure 1:**
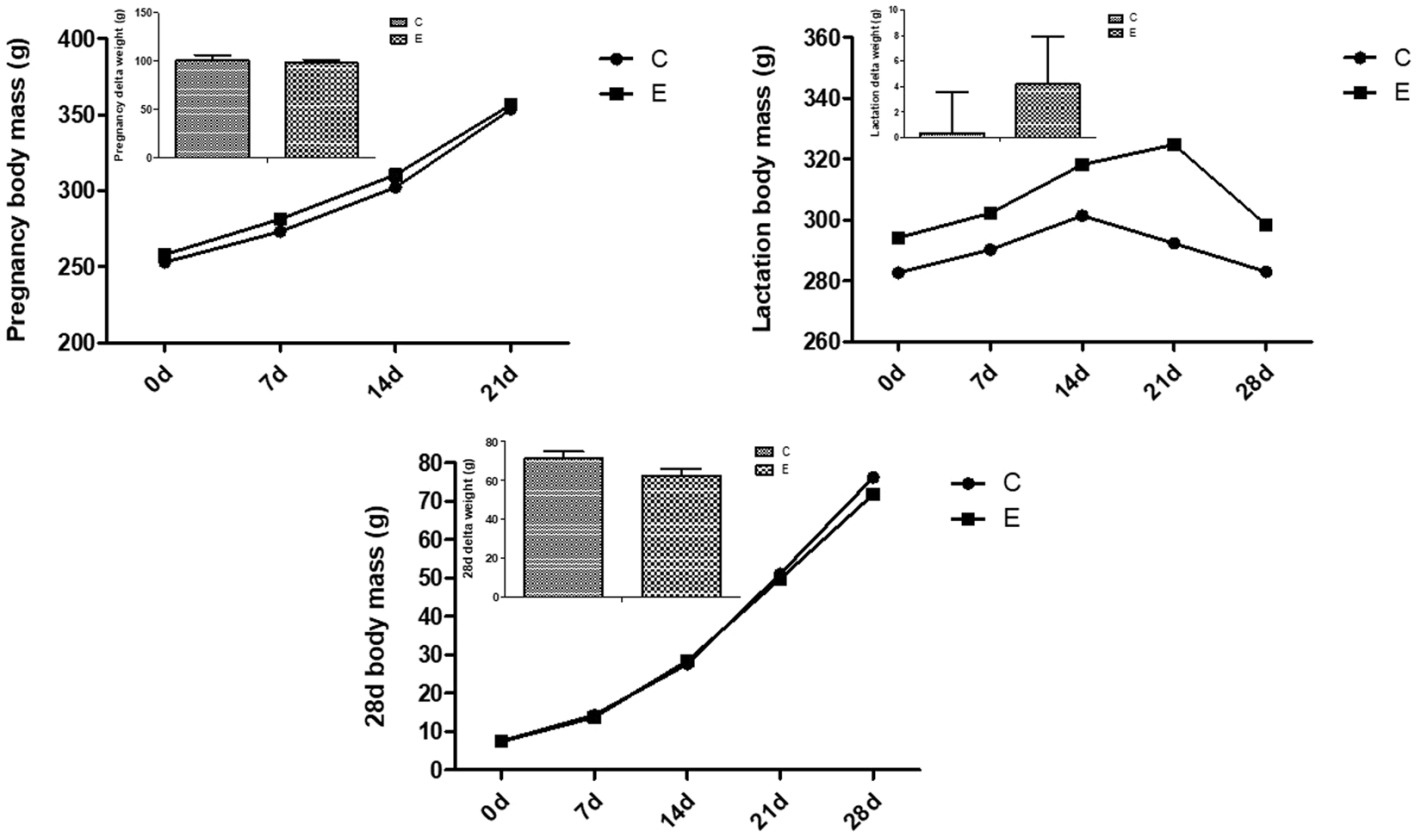
Body weight and delta weight in the different groups during all treatment. Mother – C is MC group – mother received water; Mother – E is ME group – mother received green tea extract; Pups 28d – C is FC group – pup from mother received water; Pups 28d – E is FE group – pup from mother received green tea extract. Data are mean ± SEM (n = 8–13).

The mean body weights (BWs) and delta weight of the mothers during pregnancy and lactation, and pups with 28 days old were similar among groups during all treatment (Fig. 1).

### Tissues relative weight

There was no difference in tissues relative weight in the mothers. RET relative weight in the FE group was significantly lower than in the FC (p = 0.04) group, but there were no differences in others relative weight among groups (Table 1).

**Table 1:** Relative tissue weight in different groups.

(g tissue/100 g body mass)	Mothers			Pups 28d		
	C	E	p value	C	E	p value
RET	0.89 ± 0.10	0.87 ± 0.07	0.97	0.40 ± 0.04	0.37 ± 0.02*	0.04
GON	0.87 ± 0.11	1.14 ± 0.07	0.37	0.42 ± 0.04	0.38 ± 0.03	0.54
MES	0.66 ± 0.09	0.63 ± 0.06	0.82	0.63 ± 0.03	0.57 ± 0.02	0.79
LIVER	3.87 ± 0.15	3.69 ± 0.07	0.13	3.84 ± 0.17	3.62 ± 0.11	0.27
SAT (Sum adipose tissue)	2.43 ± 0.28	2.65 ± 0.17	0.48	1.46 ± 0.09	1.32 ± 0.04	0.24

Mother – C is MC group – mother received water; Mother – E is ME group - mother received green tea extract; Pups 28d – C is FC group – pup from mothers received water; Pups 28d – E is FE group – pup from mothe received green tea extract. Data are mean ± SEM (n = 8–13); *p < 0.05, versus control group of the respective treatment.

### Serum concentrations

Serum concentrations of total cholesterol, triacylglycerol, glucose, insulin, adiponectin/SAT and HOMA-IR of the mothers and pups at 28d-old were similar among the groups. However, the serum concentration of adiponectin and LPS were significantly higher in the FE (p = 0.01 and p = 0.04, respectively) group compared with the FC group and were not different in mothers (Table 2).

**Table 2:** Cholesterol, triacylglycerol, glucose, insulin, adiponectin, LPS, adiponectin/SAT and HOMA-IR concentrations in the different groups.

	Mothers			Pups 28d		
	C	E	p value	C	E	p value
Cholesterol (mg/dL)	72.43 ± 7.32	67.72 ± 5.57	0.67	85.50 ± 6.79	93.75 ± 9.84	0.10
Triacylglycerol (mg/dL)	106.49 ± 5.24	116.68 ± 7.13	0.13	142.00 ± 11.21	159.75 ± 10.40	0.82
Glucose (mg/dL)	109.87 ± 2.54	109.39 ± 2.21	0.77	111.62 ± 7.22	109.25 ± 4.44	0.20
Insulin (ng/mL)	1.05 ± 0.30	0.85 ± 0.14	0.09	0.33 ± 0.07	0.18 ± 0.02	0.13
Adiponectin (ng/mL)	2.08 ± 0.11	2.18 ± 0.90	0.70	2.15 ± 0.03	2.34 ± 0.15*	0.01
LPS (EU/mL)	9.64 ± 2.76	8.17 ± 0.98	0.21	5.01 ± 0.14	9.36 ± 2.49*	0.04
Adiponectin/SAT	0.34 ± 0.08	0.30 ± 0.04	0.33	1.50 ± 0.10	1.78 ± 0.15	0.49
HOMA-IR	7.02 ± 2.19	5.68 ± 1.10	0.17	2.21 ± 0.49	1.24 ± 0.18	0.08

Mother – C is MC group – mother received water; Mother – E is ME group – mother received green tea extract; Pups 28d – C is FC group – pup from mother received water; Pups 28d – E is FE group – pup from mother received green tea extract. Data are mean ± SEM (n = 8–13); *p < 0.05, versus control group of the respective treatment.

### Tissues cytokine content

**Figure 2:**
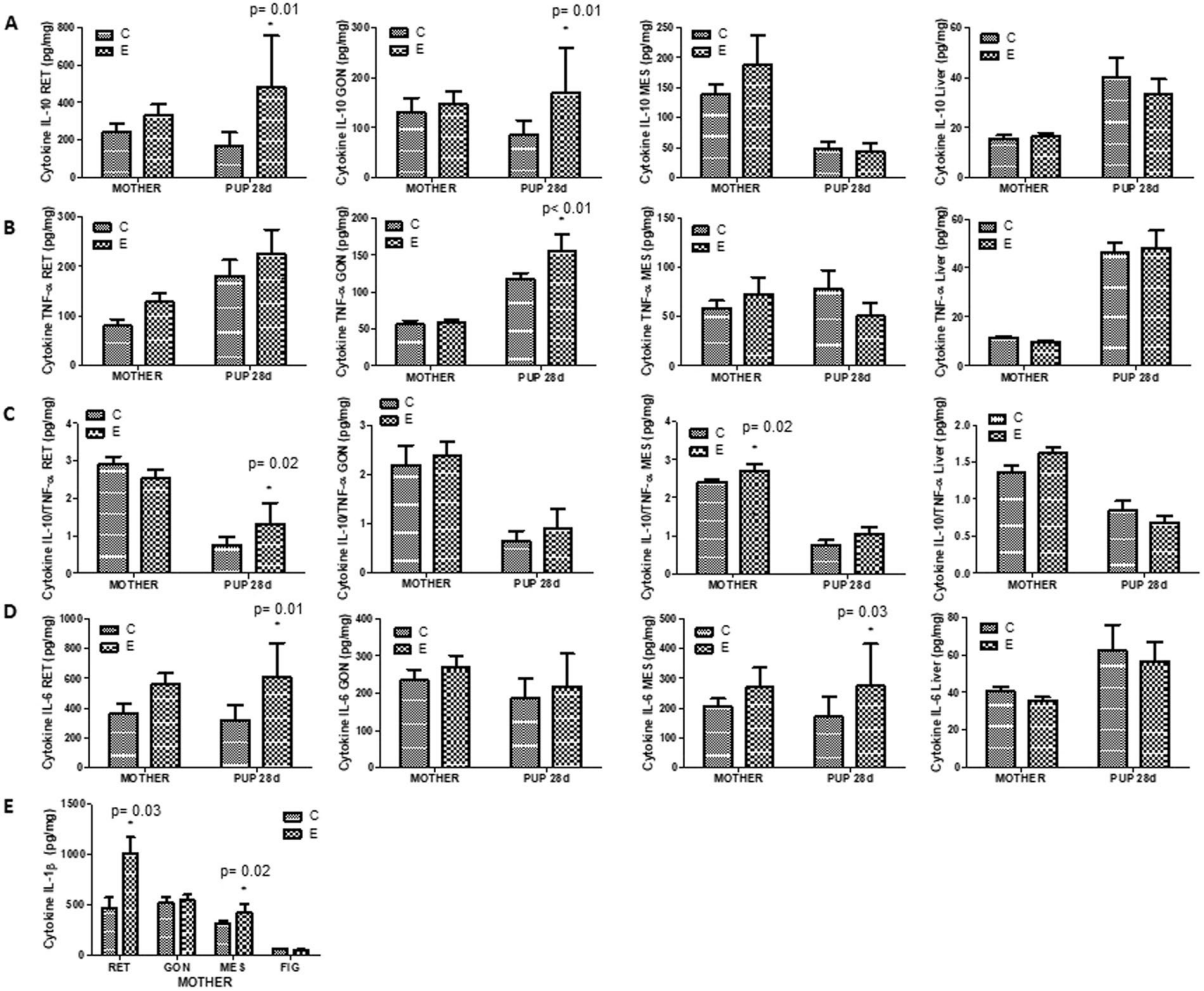
Tissues cytokine content. (**A**) IL-10 content in RET, GON, MES and Liver; (**B**) TNF-α content in RET, GON, MES and Liver; (**C**) IL-10/TNF- α content in RET, GON, MES and Liver; (**D**) IL-6 content in RET, GON, MES and Liver and (**E**) IL-1β content in RET, GON, MES and Liver. Mother – C is MC group – mother received water; Mother – E is ME group – mother received green tea extract; Pups 28d – C is FC group – pup from mother received water; Pups 28d – E is FE group – pup from mother received green tea extract. Data are means ± SEM (n = 8–13). *p < 0.05, versus control group of the respective treatment.

Figure 2A shows that the IL-10 content in RET and GON was greater in the FE group than in the FC (p < 0.01 and p = 0.01, respectively) group. In other tissues, the IL-10 content was not significantly different among the MC versus ME groups.

TNF-α content in the GON was higher in the FE group than in the FC (p < 0.01) group. In other tissues, TNF-α content did not differ among the groups (Fig. 2B).

IL-10/TNF-α ratio in the MES of the ME group was higher than in the MC (p = 0.02) group. IL-10/TNF-α ratio was higher in RET of the FE group than FC (p = 0.02) group, but not different in any other tissues or groups (Fig. 2C).

IL-6 content in RET and MES was higher in the FE group than the FC (p = 0.01 and p = 0.03, respectively) group. In the others tissues and groups did not different (Fig. 2D).

In Fig. 2E, we observed that IL-1β content in RET and MES was higher in the ME group than in the MC (p = 0.03 and p = 0.02, respectively) group.

### Antioxidant enzymes: superoxide dismutase, glutathione peroxidase and catalase

Table 3 showed the ME has lower catalase than the MC (p = 0.04) group. FE decrease the SOD when compared to the FC (p = 0.045) group. The GPx not differ in the groups.

**Table 3:** Antioxidante enzymes activity.

(units/mg protein)	Mothers			Pups 28d		
	C	E	p value	C	E	p value
SOD	6833.50 ± 409.81	6245.80 ± 173.31	0.21	1356.39 ± 255.30	596.14 ± 80.88*	0.04
GPx	0.111 ± 0.14	0.131 ± 0.02	0.12	0.31 ± 0.062	0.23 ± 0.056	0.68
Catalase	27.34 ± 1.41	23.32 ± 2.38*	0.04	159.36 ± 42.01	104.91 ± 15.22	0.09

Mother – C is MC group – mother received water; Mother – E is ME group – mother received green tea extract; Pups 28d – C is FC group – pup from mother received water; Pups 28d – E is FE group – pup from mother received green tea extract. Data are mean ± SEM (n = 8–13); *p < 0.05, versus control group of the respective treatment.

## Discussion

In the present study, maternal consumption of green tea extract during pregnancy and lactation did not promote alteration in body weight and delta weight in mothers and pups. However, mothers that consumed green tea extract demonstrated an increase in IL-10/TNF-α ratio and IL-1β in the mesenteric adipose tissue and a decrease in catalase. The 28 days old pups born from the dams which consumed green tea extract had lower relative mass of the retroperitoneal adipose tissue and lower superoxide dismutase, as well as higher adiponectin and LPS concentrations and inflammatory cytokines in adipose tissue. These results indicate that the consumption of the green tea extract by mothers altered the inflammatory status of mothers and offspring.

The animals did not present difference in body weight and delta weight associated with green tea consumption. Previous studies from our group demonstrated similar results when comparing normolipidic diet ingestion with or without green tea extract in other periods of life^13,14^. However, other studies observed reduction in body weight or delta weight when compared the consumption of green tea extract with control diet and water with control diet^15,16^. It is important to remember that in this study the offspring did not drink green tea extract.

The maternal intake of green tea extract promoted decreased in retroperitoneal adipose tissue relative weight in 28d-old pups. Cunha *et al*.^15^ demonstrated reduction in retroperitoneal adipose tissue relative weight in group that received control diet and green tea extract (400 mg green tea commercial extract/kg body weight/day) during 8 weeks, in mice. Other study with mice that received green tea extract (50 mg/kg body weight/day) and control diet per 8 weeks presented decrease in epididymal adipose tissue relative weight and SAT^17^. At this point it is interesting to note that the catechins concentration varies in different green tea extract depending on the types and the extraction time^18^ and the extract used in the current study had lower EGCG concentration, when compared to the others.

A study from our group evaluated the ingestion of 10% of prebiotic during pregnancy and lactation on 21d-old offspring and identified reduction in RET when compared to the pups from mothers that not ingested prebiotic, both with control diet^19^. Altogether, these data demonstrated the influence of maternal diet during pregnancy and lactation on pups’ metabolism.

The consumption of 400 mg/kg body mass/day of green tea extract during 8 weeks with control diet promoted increase in adiponectin serum of adult mice^15^. In another study using mice treated with green tea extract (50 mg/kg body mass/day) during 16 weeks, the authors observed an increase in adiponectin serum levels in animals fed with control diet and green tea extract^20^. In the present study, we demonstrated an increase in adiponectin serum levels in 28d-old pups demonstrating the effect of green tea consumption by mothers on pups’ metabolism.

We showed the decrease in catalase in the ME group and SOD in the FE group. Green teas possess high content of catechin, especially EGC and EGCG, and they display the highest antioxidant activity scavenging reactive oxygen species (ROS) by generating more stable phenolic radicals^21,22^. The antioxidant action of catechins in green tea is likely involved in their anti-obesity mechanism owing to the fact that ROS stimulate NFκB, which in turn promotes the expression of proinflammatory cytokines, such as TNF-α and IL-1β^23,24^.

Studies with green tea extract showed increased IL-10 and TNF-α levels in retroperitoneal, gonadal and mesenteric adipose tissue in adult mice when associated with normolipidic diet^15,17^. However, another study showed anti-inflammatory effects of the EGCG and EGC by the inhibition of IL-1β-induced soluble mediators (IL-6 and IL-8), in activated synovial fibroblasts of humans with rheumatoid arthritis^25^. We observed an increase in IL-10 levels in gonadal and retroperitoneal adipose tissue and TNF-α in gonadal adipose tissue in FE group, and additionally, an increase in IL-10/TNF- α ratio in retroperitoneal adipose tissue in FE group and in mesenteric adipose tissue in ME group. IL-6 in RET, and IL-1β in MES was increased in FE and ME group, respectively. These data demonstrate a proinflammatory effect of maternal consumption of the green tea during pregnancy and lactation in mothers and pup 28d-old.

The increased levels of TNF-α are associated with some inflammatory disorders, and IL-10 is secreted by activated monocytes/macrophages and lymphocytes and is known to possess multifaceted anti-inflammatory properties^26^. Therefore, suppressing chronic inflammation may be a good strategy to prevent and/or treat obesity that is developed by chronic systemic inflammation. Interestingly, previous studies suggest that the positive impacts of green tea polyphenols could be via their ability to suppress chronic inflammation^27–29^.

The white adipose tissue depots are subdivided into subcutaneous (for example: intramuscular and inguinal) and visceral (for example: retroperitoneal, mesenteric and gonadal) adipose tissue^30^. The visceral adipose tissue is considered a pro-inflammatory endocrine tissue and is associated with hypertension, dyslipidemia, inflammation, glucose intolerance, insulin resistance and obesity^31,32^. Previously, it was described that the obese adipose tissue is characterized by production and secretion of inflammatory molecules like TNF-α and IL-6, which may have local and systemic effects^33–35^. Also, the literature shows that LPS induced the activation of the Toll-like receptor 4 (TLR) leading to the production of proinflammatory cytokines, as IL-1β, IL-6 and TNF-α^36,37^. This study demonstrates that the ingestion of green tea extract during pregnancy and lactation promoted alteration in inflammatory response on maternal and offspring visceral adipose tissue, specific retroperitoneal and mesenteric depots.

Additionally, in the offspring from mothers that received green tea extract, the alterations on the visceral adipose tissue inflammatory milieu were accompanied by increased concentration of LPS on serum, contributing to the proinflammatory state observed in visceral adipose tissue of the pups with 28d-old.

Although it was not evidenced statistically, by analyzing the values of cytokine in the different white adipose tissue depots, we observed that the retroperitoneal adipose tissue of the mothers and offspring of the green tea group presented higher values when compared to the other groups. It seems that different visceral white adipose tissue depots could respond distinctly depending on the nutritional interference.

In summary, the maternal consumption of green tea extract associated with control diet ingestion during pregnancy and lactation promoted proinflammatory status in mothers and 28d-old offspring. These data demonstrated alterations in metabolism development of the offspring by modification in inflammatory milieu in different deposits of visceral adipose tissue. Further studies are needed in order to examine the dose-dependent effects of the green tea extract on metabolic programming.

## Methods

### Animals and treatments

The Experimental Research Ethic Committee of the Universidade Federal de São Paulo approved all procedures for the care of the animals used in this study (CEUA n°: 718008/2013). The rats were kept under controlled conditions of light (12-h light/12-h dark cycle with lights on at 07:00) and temperature (24 ± 1 °C). Three-month-old female Wistar rats were left overnight to mate, and copulation was verified the following morning by the presence of sperm in vaginal smears.

On the first day of pregnancy, the dams were isolated in individual cages, receiving control diet and were randomly divided into two groups: water (MC group) and green tea extract (ME group). The treatment was maintained throughout pregnancy and lactation.

On the day of delivery, considered day 0 of lactation, litter sizes were adjusted to nine pups each. The animals were weighed weekly.

After lactation, at day 28^th^ post-partum, the mothers and one pup from each mother were euthanized and composed the groups: FC – pup from mother received water and FE – pup from mother received green tea extract.

The green tea extract was offered in amber bottle daily at the concentration of 400 mg/kg body weight/day diluted in the water according to the volume ingested in the previous day. Composition of catechins of the green tea extract used in this study was: 16 μg/mg catechin, 29 μg/mg epicatechin, 24 μg/mg epicatechin gallate, 40 μg/mg epigalocatequinagalate and 58 μg/mg epigallocatechin.

The control diet was modified according to the recommendations of the American Institute of Nutrition (AIN- 93 G)^38^. The composition of 1 kg of diet is cornstarch (629.5 g), casein (200 g), soybean oil (70 g), fiber (50 g), vitamin mix (10 g), mineral mix (35 g), L-cysteine (3 g), choline bitartrate (2.5 g) and tert-butylhydroquinone (0.014 g). The calories percentage of the macronutrients are 63.8% carbohydrates, 20.3% protein and 16% lipids, and the energy values are 3.9 kcal/g.

### Experimental procedures

The mothers and pups were decapitated on postnatal 28 days, where 8–13 animals per group were used in the post coming analyzes. The mothers were fasted but not the 28d-old pups to avoid the weaning stress. Trunk blood was collected and immediately centrifuged. The serum was separated and stored at −80 °C for later determinations. The retroperitoneal (RET), mesenteric (MES) and gonadal (GON) white adipose tissue and liver were isolated, weighed, immediately frozen in liquid nitrogen and stored at −80 °C.

### Biochemical and hormonal serum analyses

Serum glucose, total cholesterol and triacylglycerol concentrations were measured using a commercial enzymatic colorimetric kit (Labtest®, Brazil). Insulin (Millipore®, USA), adiponectin (AdipoGen Life Sciences®), and lipopolysaccharideo (LPS) (Lonza®) concentrations were quantified using specific commercial kits. HOMA-IR and adiponectin/SAT ratio were calculated. SAT is the index of adiposity and it was calculated by the sum of MES, RET, and GON relative weight.

### IL-10, TNF-α, IL-6 and IL-1β protein concentration determined by ELISA

Following decapitation, portions of the adipose tissue (0.3 g) and liver (0.1 g) were homogenized in 800 µL of chilled extraction buffer (100 mM Trizma Base pH 7.5; 10 mM EDTA; 100 mM NaF; 10 mM Na_4_P_2_O_7_; 10 mM Na_3_VO_4_; 2 mM PMSF; 0.1 mg/ml aprotinin). After homogenization, 80 μl of 10% Triton X-100 was added to each sample. These samples were held on ice for 30 minutes and then centrifuged (20817 g, 40 minutes, 4 °C). The supernatant was collected, and protein concentrations were determined using the Bradford assay (Bio-Rad®, Hercules, California) with bovine serum albumin as a reference. Quantitative assessment of IL-10, TNF-α, IL-6 and IL-1β proteins was carried out using ELISA (DuoSet ELISA, R&D Systems, Minneapolis, MN, USA) following the recommendations of the manufacturer.

### Antioxidant enzymes activity

The liver was weighted and homogenized in specific buffer. Superoxide dismutase (SOD) and glutathione peroxidase (GPx) enzyme activities in the serum were determined using RANSOD and RANSEL Kits (Randox Laboratories, Crumlin, UK), respectively. For the catalase activity the hydrogen peroxide consumption method was used^39^. The protein concentration was measured by the Bradford method.

### Statistical analysis

All results were presented as the means ± standard error of the mean (SEM). The statistical significance of the differences between the means of the samples of the groups (MC versus ME; and FC versus FE) was assessed using independent test-t. Differences were considered to be significant when p < 0.05.
